# Gambogenic Acid Induces Endoplasmic Reticulum Stress in Colorectal Cancer via the Aurora A Pathway

**DOI:** 10.3389/fcell.2021.736350

**Published:** 2021-10-06

**Authors:** Cheng Liu, Jiaxin Xu, Chenxu Guo, Xugang Chen, Chunmei Qian, Xing Zhang, Pinghong Zhou, Yifu Yang

**Affiliations:** ^1^Experiment Center for Science and Technology, Shanghai University of Traditional Chinese Medicine, Shanghai, China; ^2^Endoscopy Center and Endoscopy Research Institute, Zhongshan Hospital, Fudan University, Shanghai, China; ^3^Eastern Hepatobiliary Surgery Hospital, Shanghai, China

**Keywords:** gambogenic acid, endoplasmic reticulum stress, Aurora A, colorectal cancer, eukaryotic translation initiation factor 2α

## Abstract

Colorectal cancer (CRC) is one of the most common malignancies in the world and has a poor prognosis. In the present research, gambogenic acid (GNA), isolated from the traditional Chinese medicine gamboge, markedly induced apoptosis and inhibited the proliferation of CRC *in vitro* and *in vivo*. Furthermore, GNA triggered endoplasmic reticulum (ER) stress, which subsequently activated inositol-requiring enzyme (IRE) 1α and the eukaryotic translation initiation factor (eIF) 2α pathway. Pretreatment with salubrinal (an eIF2α inhibitor) rescued GNA-induced cell death. Furthermore, GNA downregulated the expression of Aurora A. The Aurora A inhibitor alisertib decreased ER stress. In human colorectal adenocarcinoma tissue, Aurora A was upregulated compared to normal colorectal epithelial nuclei. Furthermore, GNA ameliorated mouse colitis-associated cancer models. Our findings demonstrated that GNA significantly inhibited the proliferation of CRC through activation of ER stress by regulating Aurora A, which indicates the potential of GNA for preventing the progression of CRC.

## Introduction

Colorectal cancer (CRC) is the most common malignant tumour of the gastrointestinal tract and has become the third most common malignant tumour worldwide (Keum and Giovannucci, 2019). The majority of patients discover disease after local or distant metastasis and miss opportunities for surgical treatment (Hosseini et al., 2016; Siegel et al., 2017). The treatment of advanced stage CRC is mainly adjuvant chemotherapy, but common chemotherapy regimens have obvious side effects. The 5-year survival rate ranges from 90% of patients with localised disease to more than 14% of patients with distant-stage disease (Siegel et al., 2020). Despite tremendous progress in medical treatments, the influence of these treatments on the cure rate and long-term survival rate of CRC is limited. Therefore, the development of novel CRC drugs is urgently needed.

The endoplasmic reticulum (ER) is an important organelle for protein synthesis, processing, modification and transport. Abnormal ER stress responses are associated with many diseases, including cardiovascular disease (Ochoa et al., 2018), genetic skeletal diseases (Briggs et al., 2020), and cancer (Chipurupalli et al., 2019). Multiple studies have reported that ER stress plays a proapoptotic role in CRC (Albayrak et al., 2021; Zhao et al., 2021). The ER stress response is the result of the excessive accumulation of unfolded and misfolded proteins in the ER. The high proliferation rate of cancer cells leads to ER stress, and the cytoprotective mechanism of the unfolded protein response (UPR) is triggered to establish ER homeostasis. The UPR is promoted to convert into apoptotic machinery (Delbrel et al., 2018; Kim and Kim, 2018), which is controlled by the PKR-like ER kinase (PERK), IRE1α and activating transcription factor (ATF) 6 (Urra et al., 2016) pathways. ER stress persistence or worsening results in the failure to establish ER homeostasis, and the effect of ER stress is transformed from pro-survival to pro-apoptotic (Kim and Kim, 2018). ER stress is a target for anticancer agents, for example, ruthenium, palladium and platinum complexes, which are ER-targeting anticancer agents for the treatment of cancer, possess tremendous potential (King and Wilson, 2020).

The Aurora kinase family consists of three serine/threonine kinases, including Aurora A, B, and C, which are essential kinases for cell division through the regulation of mitosis (Joukov and De Nicolo, 2018). The expression of Aurora A is regulated by the cell cycle, and activation of Aurora A in the late G2 phase of the cell cycle is required for mitotic entry (Shen et al., 2019). Aurora A promoted glycolysis through phosphorylation of lactate dehydrogenase B (Cheng et al., 2019) and enhanced drug resistance by stabilising forkhead box protein M1 (Yang et al., 2019). Aurora A is a therapeutic target and a poor prognostic factor in various tumours, such as lung cancer (Wang J. et al., 2021), adrenocortical cancer (Maria et al., 2021) and breast cancer (Jalalirad et al., 2021). Therefore, Aurora A inhibitors possess good prospects for clinical application (Yan et al., 2016).

Gambogenic acid (GNA), which is the main active component of gamboge, is a derivative of gambogic acid. In recent years, GNA has been a potential anticancer candidate due to its anticancer activities. GNA inhibits a variety of cancers, such as lung cancer, breast cancer and colorectal cancer (Yan et al., 2012; He et al., 2015; Zhao et al., 2020). The anticancer effects of GNA are associated with the induction of apoptosis (Chen et al., 2017) and autophagy (Mei et al., 2014) and the enhancement of chemosensitivity (He et al., 2015). Previous studies have reported that GNA induces apoptosis via mitochondrial dependence (Yan et al., 2012) and death receptors (Zhou et al., 2013). Although it has been reported that GNA triggers ER stress-mediated apoptosis through the reactive oxygen species/IRE1α/c-Jun N-terminal kinase signalling pathway in CRC (Zhao et al., 2020) and triggers apoptosis via heavy chain-binding protein (Bip), ATF4 and C/EBP-homologous protein (Chop) in nasopharyngeal carcinoma (Su et al., 2019), the mechanism of GNA-induced ER stress is still incomplete. In the present study, we will deeply investigate the effects of GNA-induced ER stress on CRC *in vitro* and *in vivo*.

## Materials and Methods

### Animals and Material Preparation

Male BALB/c mice (8–10 weeks) were purchased from Shanghai Laboratory Animal Center of the Chinese Academy of Sciences and raised by the Animal Experiment Center of Shanghai University of Traditional Chinese Medicine. All animal experiments were conducted in accordance with the procedures approved by the Animal Care and Use Committee of Shanghai University of Traditional Chinese Medicine.

HCT116 and HT29 cells were purchased from Sailybio (Shanghai, China). RPMI 1640, penicillin and streptomycin, fetal bovine serum (FBS), BSA, ER-tracker TM Green and Lipofectamine^TM^ 2000 Transfection Reagent were purchased from Invitrogen (Carlsbad, CA, United States). AOM, thapsigargin, salubrinal and 4-PBA were obtained from Sigma-Aldrich (St. Louis, MO, United States). PrimeScript^TM^ RT Master Mix, TB Green Premix Ex Taq^TM^ and RNAiso Pius were purchased from TaKaRa (Tokyo, Japan). GSK2606414 and Alisertib were purchased from Selleckchem (Houston, TX, United States). DSS was purchased from MP Biomedicals (Santa Ana, CA, United States). PVDF membranes were purchased from Millipore (Billerica, MA, United States).

### Cell Culture

Cells were cultured with RPMI 1640 medium supplemented with 10% foetal bovine serum, 100 U/ml penicillin and 100 μg/ml streptomycin and cultivated at 37°C in an incubator supplying 5% CO_2_.

### MTT Assay

HCT116 cells were seeded in a 96 well microplate at 4,000 cells/well. GNA was added and incubated for 24 h. MTT was added to the well and incubated for 4 h. After discarding the supernatant, the precipitate was dissolved in DMSO. The absorbance of each well was measured at 570 nm.

### Cell Cycle Assay

HCT116 cells were treated with the indicated concentrations of GNA for 24 h. After washing with PBS, the cells were detached by trypsinization and fixed in 70% ethanol overnight at 4°C. The cells were then washed with PBS, and reacted with 20 μg/ml propidium iodine (PI) in the dark for 10 min. The cell cycle distribution was measured using a FACSCalibur (BD, New Jersey, United States).

### Endoplasmic Reticulum Tracker Staining

HCT116 cells were treated with GNA for 6 h in glass bottom cell culture dishes, and the cells were incubated with ER-tracker TM Green (1 μM) at 37°C for 30 min. Then, images of ER fluorescence were taken by confocal laser scanning microscopy (Leica, Wetzlar, Germany).

### Transmission Electron Microscopy

The TEM was performed as previously described (Wang X. et al., 2021). Briefly, the cells were fixed with 2.5% glutaraldehyde in PBS for 1 week, followed by 2% OsO_4_. After dehydration, the sections were stained with 3% uranyl acetate and lead citrate for observation under TEM.

### Western Blot Analysis

Total protein was extracted from HCT116 cells in RIPA buffer. A BCA kit was applied to measure the protein concentration. Then, proteins were electrophoresed on SDS-PAGE gels and transferred onto PVDF membranes. After non-specific blocking with BSA for 1 h, the membranes were probed with primary antibodies. Then, the membrane was incubated with secondary antibodies at room temperature for 1 h. The signals were captured through ECL Blotting Detection Reagents. The following primary antibodies were used in this study: Bip (CST#3177); IRE1α (CST#3294); PERK (CST#5683); p-PERK (Affinity#DF7576); p-eIF2α (CST#3398); eIF2α (CST#5324); ATF4 (CST#11815); Aurora A (CST#14475); and GAPDH (CST#2118).

### Real-Time PCR Analysis

Total RNA was extracted with RNAiso Pius following the manufacturer’s protocol. Then, cDNA was reverse transcribed using PrimeScript^TM^ RT Master Mix. Quantitative detection of mRNA was performed through real-time PCR (Applied Biosystems, Foster, CA, United States). The sequences of the primers used are in Supplementary Table 1.

### Microfluidic Mobility Shift Enzyme Assay

The mobility shift enzyme assay was performed as previously described (Xu et al., 2018). Briefly, the compound diluent was mixed with the substrate, enzyme was added to incubate at room temperature for 10 min. Then, the reaction was stopped by a stop buffer. The enzyme conversion data were then readout for analysis.

### Colitis-Associated Cancer (CAC) Model

The CAC model was assessed as previously described (Hayashi et al., 2014). Mice were administered AOM (12 mg/kg) intraperitoneally. Five days later, mice were administered 2% DSS for 5 days, followed by 16 days of regular water. This cycle was repeated a total of three times. On day 100 after AOM administration, colons were removed from mice, flushed with cold PBS, and fixed in 10% paraformaldehyde at room temperature overnight.

### H&E Staining

The tissues were fixed in 4% paraformaldehyde and embedded in paraffin. Then, paraffin blocks were sectioned with a slicer at a distance of 4 μm. Sections were stained with haematoxylin and eosin.

### Immunohistochemistry (IHC)

Paraffin blocks were sectioned and stained with anti-Bip and anti-Chop antibodies overnight at 4°C. The slides were incubated with anti-rabbit antibody for 45 min at 37°C, developed in 0.05% freshly prepared diaminobenzidine solution for 8 min, counterstained with haematoxylin, and dehydrated.

### Immunohistochemistry on Tissue Microarrays (TMAs)

Colorectal cancer tissue samples were collected at Zhongshan Hospital. The study was approved by the Ethics Committee of Zhongshan Hospital, Fudan University (B2018-276) in accordance with the Helsinki Declaration. Immunohistochemical staining was performed on TMAs containing 233 CRC tissue samples. For each sample, two cores from different locations were included in the array. A 4-μm section of TMAs was used for immunohistochemistry and stained with monoclonal anti-Aurora A antibody (Abcam#ab52973) as described previously (Carvalho et al., 2009). Aurora A staining was evaluated on the basis of the frequency of positive nuclei, and intensity was divided into four groups: negative (<10%), weak (≥10% and <25%), moderate (≥25% and <50%) or strong (≥50%) (Belt et al., 2012; Dotan et al., 2012). All samples were grouped by two independent observers in a blinded manner. If disagreement between the two observers occurred, a third observer was consulted, and the majority group was noted.

### Statistical Analysis

Statistical analyses were performed using GraphPad Prism 7. The data are shown as the mean ± SD. The statistical analysis was performed using Student’s *t-*test or one-way ANOVA. *P* < 0.05 was considered to be statistically significant.

## Results

### Gambogenic Acid Inhibited Cell Proliferation and Induced Apoptosis in Colorectal Cancer

**FIGURE 1:**
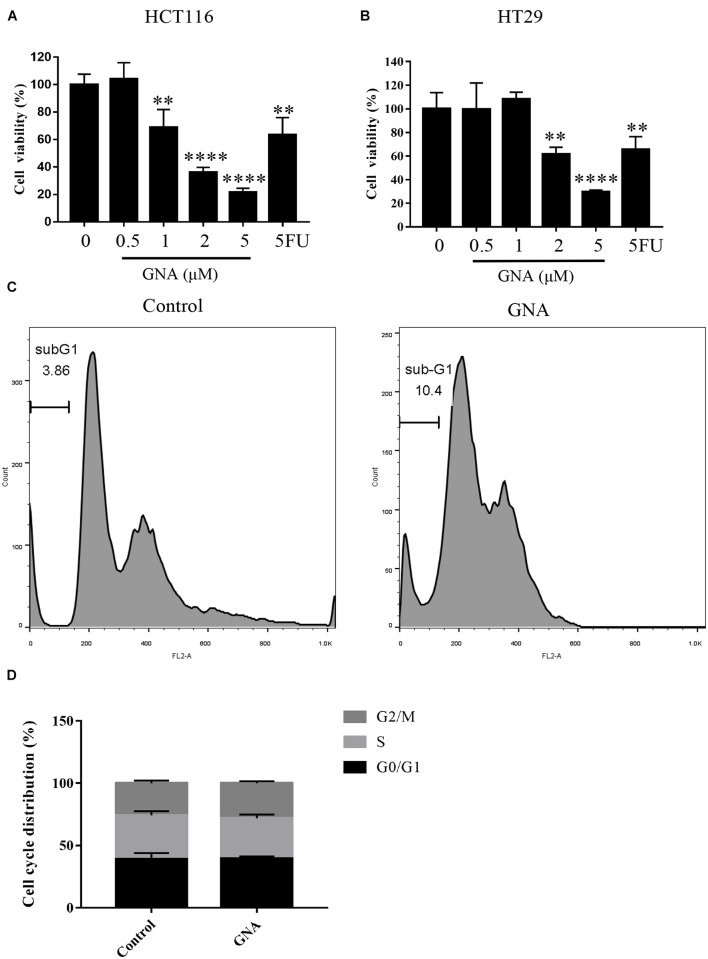
GNA inhibited cell proliferation and induced apoptosis. HCT116 **(A)** and HT29 **(B)** cell viability was assessed using the MTT assay. Cells were treated with different concentrations of GNA (0–5 μM) and 5FU (2 μM) for 24 h. MTT solution was added to the well and incubated for 4 h at 37°C. The precipitate was dissolved in DMSO, and the absorbance was measured at 570 nm. **(C,D)** Cells were treated with GNA for 24 h, and then the distribution of the cell cycle was detected by flow cytometry after PI staining. The data are presented for at least three independent experiments. The data are presented as the mean ± SD. **P < 0.01; ****P < 0.0001.

As shown in Figures 1A,B, treatment with GNA (0–5 μM) and the positive control drug 5-fluorouracil (5FU, 2 μM) for 24 h significantly reduced HCT116 and HT29 cell viability. The number of cells in sub-G1 phase was significantly increased in GNA-treated HCT116 cells compared with untreated cells (Figure 1C), suggesting that GNA can induce cell apoptosis. As shown in Figure 1D, GNA did not alter cell cycle in statistical graph. Moreover, transmission electron microscopy (TEM) data showed that GNA-treated HCT116 cells exhibited apoptosis and nuclear shrinkage (Supplementary Figure 1). Taken together, GNA inhibited CRC proliferation by inducing apoptosis.

### Gambogenic Acid Induced Apoptosis by Activating Endoplasmic Reticulum Stress

**FIGURE 2:**
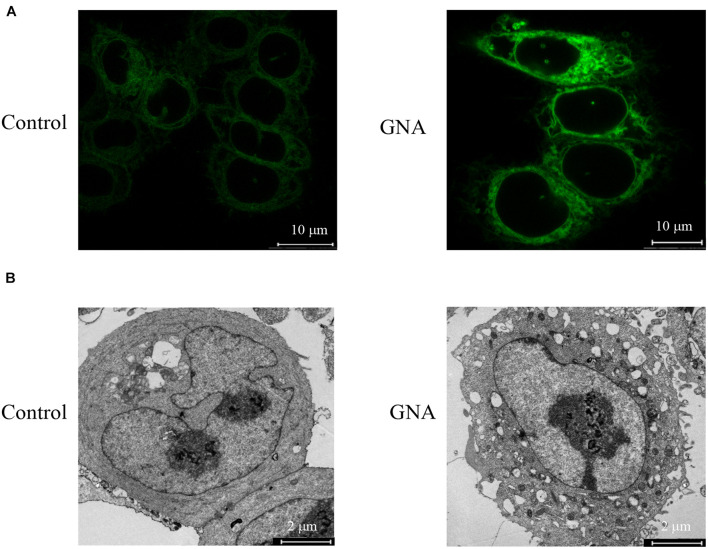
GNA activated ER stress in cells. **(A)** HCT116 cells were treated with GNA for 6 h, and then incubated with ER-tracker TM Green (1 μM) for 30 min. ER tracker staining fluorescence was observed by confocal laser scanning microscopy (scale bar = 10 μm). **(B)** HCT116 cells were treated with GNA (1 μM) for 24 h, and then the morphology of the ER was observed under TEM (scale bar = 2 μm). The data are presented for at least three independent experiments.

Abnormal ER stress responses are associated with tumour development. Thus, we investigated the GNA-induced ER fluorescence of HCT116 cells. As shown in Figure 2A, the induction of ER stress by GNA significantly increased the fluorescence intensity of the ER tracker Green, suggesting that ER stress was involved in GNA-induced apoptosis. TEM data showed that GNA-treated HCT116 cells led to swelling and ER vacuoles (Figure 2B). We considered that ER stress was induced by GNA treatment in HCT116 cells. Perturbation of intracellular calcium concentration leads to reduced protein-folding capacity of ER, which promotes ER stress (Bahar et al., 2016). Therefore, we measured the effect of GNA and thapsigargin (an intracellular calcium releaser) on calcium elevation. As shown in Supplementary Figure 2, the calcium elevation of HCT116 cells during the experimental period was significantly increased by thapsigargin; however, GNA treatment did not affect calcium, suggesting that GNA cannot directly elevate calcium concentrations.

**FIGURE 3:**
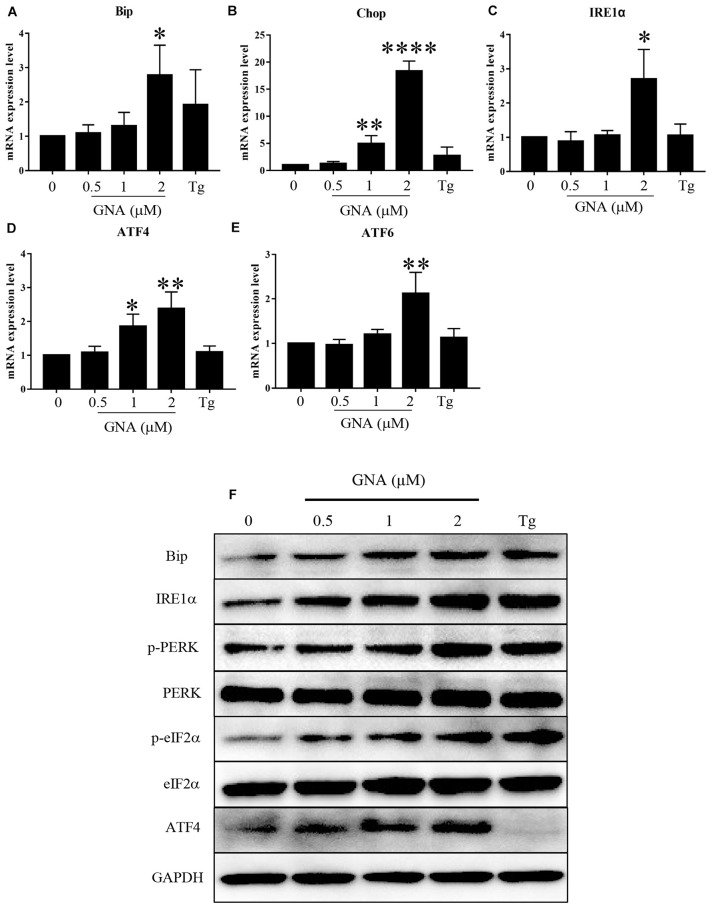
GNA promoted UPR in cells. HCT116 cells were treated with different concentrations of GNA (0.5, 1, 2 μM) and thapsigargin (1 μM) for 6 h. **(A–E)** Total mRNA was extracted, and real-time PCR was used to measure the mRNA expression levels of Bip **(A)**, Chop **(B)**, IRE1α **(C)**, ATF6 **(D)**, ATF4 **(E)**. **(F)** The expression of BIP, IRE1α, p-PERK, PERK, p-eIF2α, eIF2α, and ATF4 was analysed by western blotting. GAPDH was used as a loading control. The data are presented for at least three independent experiments. The data are presented as the mean ± SD. *P < 0.05; **P < 0.01 and ****P < 0.0001.

If ER stress worsens, heavy chain-binding protein (Bip) serves as the primary sensor in the activation of the UPR. We found that Bip mRNA (Figure 3A) was upregulated by GNA, consistent with previous studies (Su et al., 2019). As shown in Figure 3B, GNA increased the mRNA expression of Chop, a downstream target of the UPR, which can induce apoptosis (Hu et al., 2018). The UPR signal consists of IRE1α, ATF6 and PERK. We found GNA upregulated the mRNA expression of IRE1α (Figure 3C), consistent with previous studies (Zhao et al., 2020). We also found the mRNA expression of ATF4 (Figure 3D) and ATF6 (Figure 3E) was increased by GNA treatment. Similar to the results of real-time PCR, western blot outcomes revealed that GNA obviously increased Bip and IRE1α, consistent with previous studies. The PERK branch contained the activation of eIF2α and ATF4. Furthermore, we found that GNA induced not only PERK phosphorylation, but also downstream PERK phosphorylation, such as eIF2α phosphorylation and ATF4 expression (Figure 3F). Thus, we speculated that in addition to IRE1α, GNA also activated ER stress by promoting the PERK signalling pathway.

### Gambogenic Acid Promoted Endoplasmic Reticulum Stress by Targeting the eIF2α Signalling Pathway

To examine the role of the ER chaperone PERK in GNA-induced ER stress in HCT116 cells, cells were exposed to 4-phenylbutyric acid (4-PBA, a chemical ER chaperone, 2 mM) or GSK2606414 (PERK inhibitor, 10 μM), and then these cells were treated with GNA. As shown in Supplementary Figure 3, the MTT assay showed that 4-PBA or GSK2606414 failed to overcome GNA-induced cell death. We further examined the role of Bip in GNA-induced ER stress. Bip was knockdown by Bip siRNA was transfected into HCT116 cells. As shown in Supplementary Figure 4A, efficiency of Bip silencing was well. After Bip silencing and GNA treatment, we assessed the constitution of the cell cycle. As shown in Supplementary Figures 4B,C, the percentage of HCT116 cells in the sub-G1 phase was not altered. These data indicated that GNA-induced ER stress does not occur via the PERK or Bip pathway, which may be accompanied by GNA-induced cell death.

**FIGURE 4:**
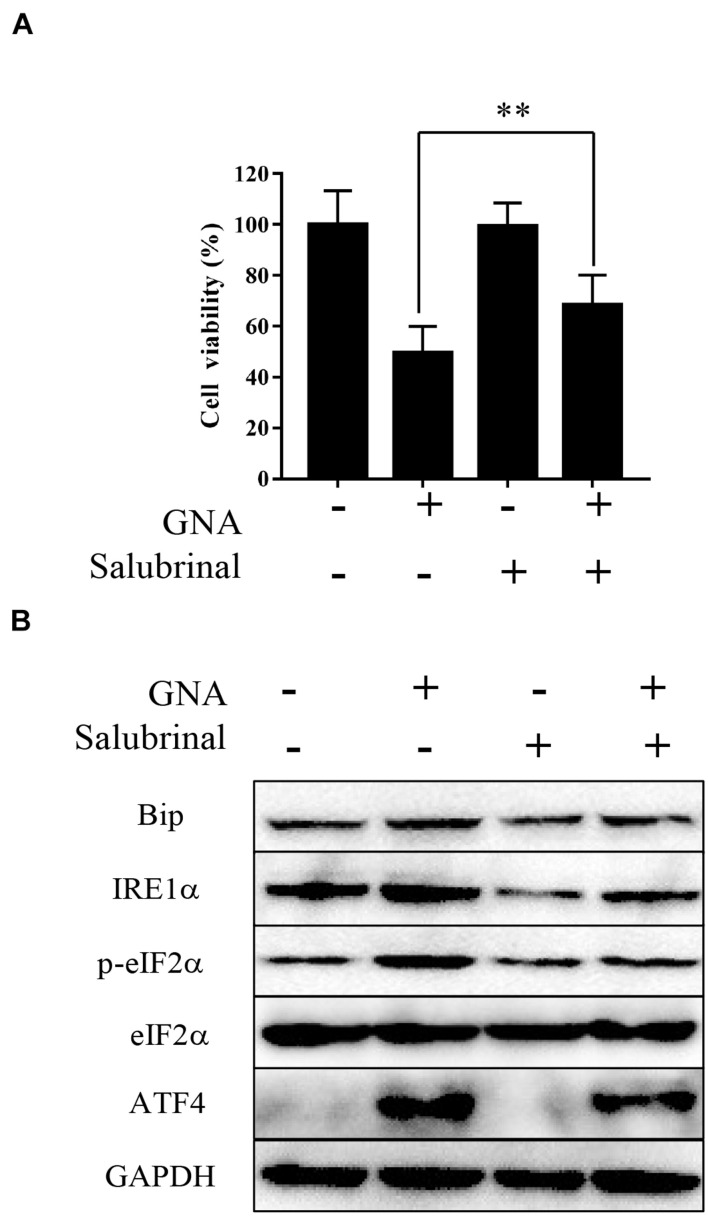
GNA activated ER stress-associated apoptosis by targeting eIF2α. HCT116 cells were treated with GNA (1 μM) after pretreatment with salubrinal (10 μM). **(A)** After GNA treatment 24 h, MTT assay was used to analyse cell viability. MTT solution was added to the well and incubated for 4 h at 37°C. The precipitate was dissolved in DMSO, and the absorbance was measured at 570 nm. **(B)** After GNA treatment 6 h, The expression of Bip, IRE1α, p-eIF2α, eIF2α, and ATF4 was analysed by western blotting. The data are presented for at least three independent experiments. **P < 0.01.

Salubrinal, an inhibitor of eIF2α phosphorylation, is an ER stress inhibitor. We further measured viability and the level of related proteins under cotreatment with GNA and salubrinal in HCT116 cells. As shown in Figure 4A, MTT showed that pretreatment with salubrinal could reverse GNA-induced cell death. Western blot analysis showed that GNA promoted the expression of Bip, IRE1α, ATF4 and phosphorylation of eIF2α, which was reversed through salubrinal treatment (Figure 4B). These data indicated that GNA induced cell death by activating ER stress to promote the phosphorylation of eIF2α.

### Gambogenic Acid Triggered Endoplasmic Reticulum Stress by Downregulating Aurora A Expression

**FIGURE 5:**
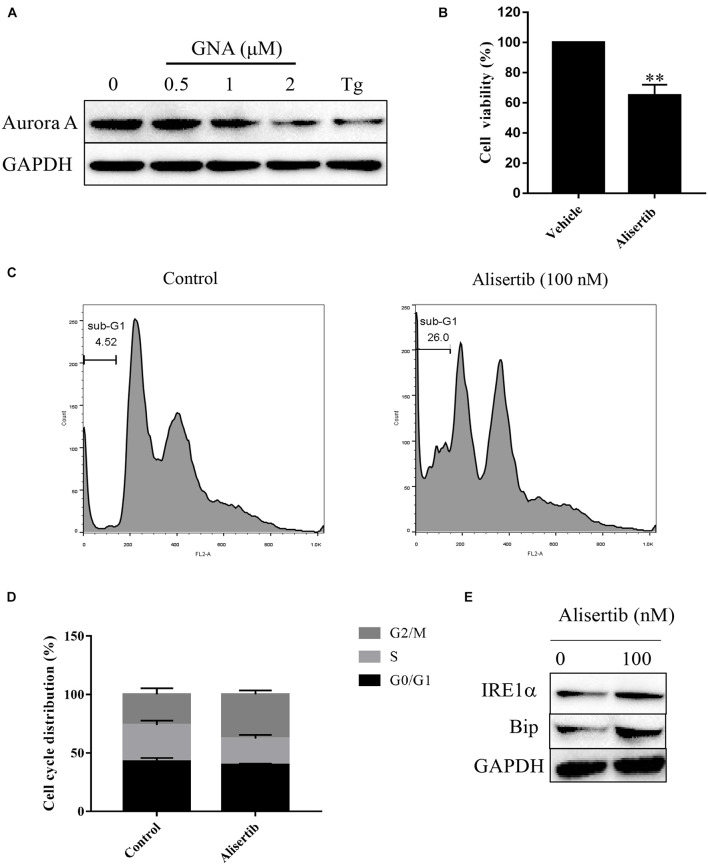
GNA activated ER stress-associated apoptosis through Aurora A. **(A)** HCT116 cells were treated with different concentrations of GNA (0.5, 1, 2 μM) and thapsigargin (1 μM) for 6 h. The expression of Aurora A was analysed by western blot. **(B)** HCT116 cells treated with alisertib (100 nM) for 24 h. MTT assay was performed to detect the suppressed proliferation effect of alisertib. **(C,D)** HCT116 cells treated with alisertib (100 nM) for 24 h. Cells were fixed in 70% ethanol at 4°C, and the cell cycle was examined by flow cytometry after PI staining. **(E)** The expression of Bip and IRE1α was analysed by western blotting. The data are presented for at least three independent experiments. The data are presented as the mean ± SD. **P < 0.01.

Furthermore, we performed a mobility shift assay to evaluate the effect of GNA on the kinases and found that GNA targeted the Aurora A kinase. GNA inhibited Aurora A, and the IC_50_ was 1.425 μM. Then, we investigated whether GNA interacts with Aurora A in cells by performing cellular thermal shift assay. As shown in Supplementary Figure 5, over the examined temperature range of 40–58°C, Aurora A levels were reduced in the GNA group compared to control group. We determined that GNA and thapsigargin both significantly downregulated the expression of Aurora A (Figure 5A), suggesting that ER stress is associated with Aurora A. It has also been reported that knockdown of Aurora A can inhibit proliferation and promote apoptosis (Zhang et al., 2021). The level of intracellular calcium was significantly increased through Aurora A inhibitor treatment (Plotnikova et al., 2011), which may induce ER stress. As shown in Figure 5B, Aurora A inhibitors (alisertib, 100 nM), a selective Aurora A inhibitor (Wang et al., 2017), inhibited the proliferation of HCT116 cells for 24 h. Moreover, cell cycle distribution was assessed by flow cytometry following PI staining. As shown in Figures 5C,D, alisertib significantly enhanced the percentage of cells in the sub-G1 phase compared to the control group, suggesting that Aurora A inhibits tumour growth, which was consistent with previous results. Then, we investigated whether Aurora A would also activate the relevant signalling pathway of ER stress. Compared with the control group, alisertib upregulated the expression of IRE1α and Bip (Figure 5E). These data showed that GNA-induced ER stress may occur through the Aurora A pathway.

### Aurora A Was Expressed in Colorectal Adenocarcinoma

**FIGURE 6:**
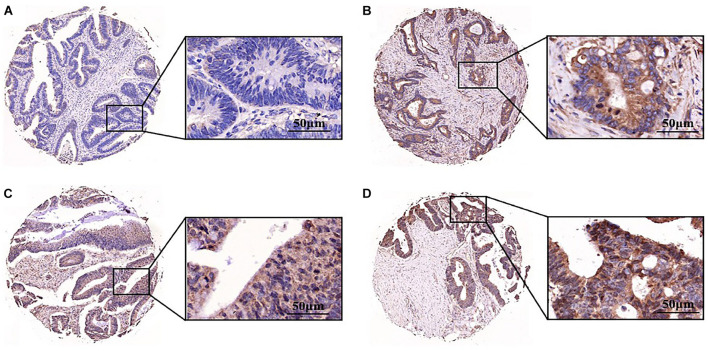
Aurora A was highly expressed in colorectal adenocarcinoma. Expression pattern of Aurora kinase A in tissue microarray cores of colorectal adenocarcinoma. The staining intensity of the nuclei was evaluated as **(A)** negative, **(B)** weak, **(C)** moderate or **(D)** strong (scale bar = 50 μm). The data are presented for at least three independent experiments.

Aurora A protein was expressed in 55.4% (129/233) of colorectal adenocarcinomas, while normal colorectal epithelial nuclei showed no evidence of protein expression. The staining pattern was nuclear and cytoplasmic (Figures 6A–D). The percentage of tumour cells positive for the protein ranged from 10 to 60%. The correlations of Aurora A expression and clinicopathological characteristics are shown in Table 1. Protein expression was closely associated with T stage (*P* = 0.042). In the T1 stage of colorectal adenocarcinoma, 14.3% (1/7) were positive for the protein, and in the T2 stage, 40% (8/20) were positive, whereas in the T3 and T4 stages, over 50% were positive, with positivity rates of 60.8% (76/125) and 54.3% (44/81), respectively. Aurora A expression was not significantly correlated with sex, age, lymphatic metastasis, distant metastasis, or TNM stage.

**TABLE 1:** The relationships between Aurora A expression and clinicopathological characteristics.

Characteristic	Aurora kinase A expression		*P* value
	Positive (*n* = 129)	Negative (*n* = 104)	
Age (year)			0.892
<65	82 (55.0)	67 (45.0)	
≥65	47 (56.0)	37 (44.0)	
Sex			0.537
Male	82 (56.9)	62 (43.1)	
Female	47 (52.8)	42 (47.2)	

**TNM stage**			

T			0.042
1	1 (14.3)	6 (85.7)	
2	8 (0.40)	12 (0.60)	
3	76 (60.8)	49 (39.2)	
4	44 (54.3)	37 (45.7)	
N			0.740
0	59 (53.2)	52 (46.8)	
1	39 (55.7)	31 (44.3)	
2	31 (59.6)	21 (40.4)	
M			0.254
0	92 (53.2)	81 (46.8)	
1	37 (61.7)	23 (38.3)	
Total			0.141
I	6 (31.6)	13 (68.4)	
II	43 (57.3)	32 (42.7)	
III	43 (54.4)	36 (45.6)	
IV	37 (52.1)	34 (47.9)	

### Gambogenic Acid Induced Endoplasmic Reticulum Stress *in vivo*

**FIGURE 7:**
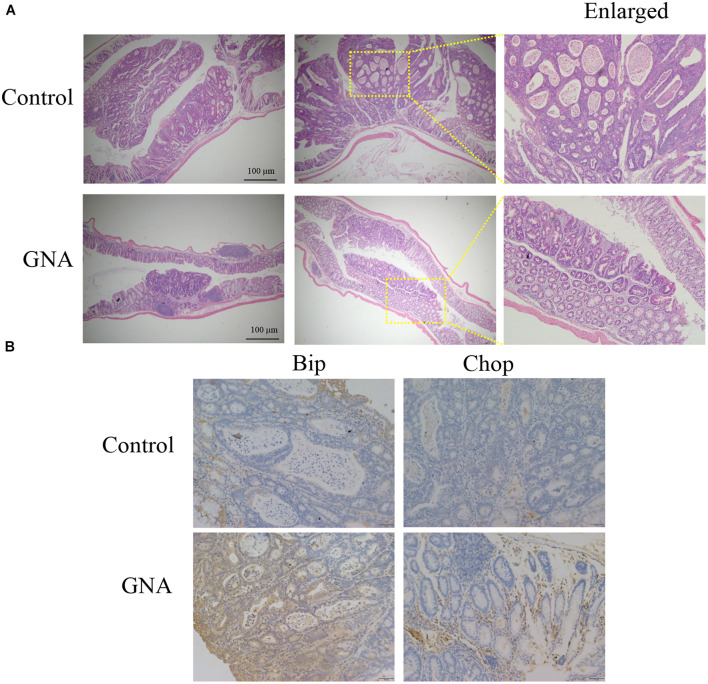
GNA inhibited CRC growth *in vivo*. An AOM/DSS mouse model of CAC was established in BALB/c mice. Colon tissue was collected after the mice were sacrificed, tissues were fixed in 4% paraformaldehyde and embedded in paraffin, and paraffin blocks were sectioned with a slicer. **(A)** The tissues were stained with haematoxylin and eosin to observe the tumour area. **(B)** Tissues were stained with anti-Bip and anti-Chop antibodies for IHC analysis. (scale bar = 100 μm). The data are presented for at least three independent experiments.

To further evaluate the antitumour effect of GNA *in vivo*, an azoxymethane (AOM)/dextran sulfate sodium (DSS) mouse model of colitis-associated cancer (CAC) was established in BALB/c mice (Supplementary Figure 6A). We found that the colon length of the control group was significantly shortened compared to that of the normal group. GNA and 5FU administration ameliorated the colon length compared to the control group (Supplementary Figure 6B). As shown in Figure 7A GNA administration suppressed colorectal tumour growth and ameliorated the altered appearance of goblet cells. In addition, as shown in Figure 7B, GNA treatment increased the expression of Chop and Bip in tumours compared with the control group, which was consistent with the *in vitro* data. However, GNA did not alter the expression of Aurora A (data not shown), indicating that the model shows a difference according to species. The above results clearly suggest that GNA suppresses CRC by triggering ER stress *in vivo*.

## Discussion

Colorectal cancer is a common malignancy, and its incidence has been on the rise in the young (Patel and Ahnen, 2018). Gambogic acid and GNA are major bioactive ingredients isolated from gamboges, and have been used in traditional Chinese medicine. GNA opens the pyran ring derivative of GA and has a broad spectrum of antitumour effects and lower toxicity. GNA remarkably inhibited the proliferation of cancer, promoted cell apoptosis and cell cycle arrest (Huang et al., 2019) and overcame cisplatin resistance (Shen et al., 2020). In the present study, we further evaluated the anticancer effects of GNA through ER stress in CRC *in vivo* and *in vitro*.

First, we demonstrated that GNA inhibited HCT116 cell proliferation and induced apoptosis. The disruption of ER homeostasis triggered the UPR, which led to cell adaptation to stress and activated IRE1, Bip and eIF2α phosphorylation. Furthermore, we found that the eIF2α inhibitor salubrinal rescued GNA-induced cell death and reduced the expression of related proteins. However, GNA did not reverse the effect of loss of Bip or PERK. Thus, we hypothesised that GNA promotes the phosphorylation of eIF2α and that downstream Chop directly or indirectly promotes apoptosis. We speculated that the eIF2α signal may play a key role in GNA-induced ER stress activation.

The molecular mechanisms of GNA mainly include regulating NF-κB signalling (Zhou et al., 2020), activating volume-sensitive outwardly rectifying chloride channels (Su et al., 2019) and increasing the level of apoptosis-related proteins (Huang et al., 2019). We found that GNA interacts with the Aurora A by shift assay, suggesting that the Aurora A signalling pathway may be a new target of GNA against CRC cells. Aurora A plays a key role in regulating the cell cycle from G2 to cytokinesis (Yan et al., 2016). It has been previously reported that AT-rich interaction domain 3A promotes the development of colorectal cancer by upregulating Aurora A (Tang et al., 2021), and the expression and prognostic values of Aurora A may be significant for CRC diagnosis (Fadaka et al., 2021). In addition, Aurora A induced mitochondrial-associated pathway apoptosis (Liu et al., 2020). We found that alisertib inhibited proliferation via ER stress-mediated apoptosis in HCT116 cells. A previous study showed that an Aurora A inhibitor increased intracellular calcium levels and disrupted the homeostasis of calcium within cells (Wang et al., 2020), which may be one of the reasons for triggering ER stress. Furthermore, we found that thapsigargin also inhibited Aurora A expression. Thus, we speculated that Aurora A was associated with the activation of ER stress. Hence, we considered that GNA suppressed CRC through ER stress by downregulating Aurora A and could be used as an Aurora A inhibitor in preclinical research.

The expression of Aurora A was a biomarker and associated with poor prognosis in patients with colorectal cancer liver metastasis (Goos et al., 2013). We assessed the expression of Aurora A in tissue samples by tissue microarrays. Aurora A protein was expressed in more than 50% of tumour tissue, while normal colorectal epithelial nuclei were not expressed. We found that Aurora A expression was significantly associated with primary tumours. Our work suggests that Aurora A may be a potential therapeutic target for the treatment of CRC and provides a new strategy for diagnosis and prognosis.

Furthermore, we analysed the antitumour effect of GNA *in vivo*. We found that GNA suppressed tumour growth by triggering ER stress *in vivo*. However, GNA treatment did not inhibit the expression of Aurora A, which is not consistent with the *in vitro* data. This difference might be related to HCT116 cells being isolated from human primary colon carcinoma, while a mouse model of CAC was established; this is an area worthy of further study.

In conclusion, our results demonstrated a novel target and molecular mechanism of the natural product GNA. These findings indicated that GNA inhibited CRC proliferation by activating ER stress *in vitro* and *in vivo*. Moreover, GNA triggered ER stress by regulating Aurora A, which could be a novel mechanism underlying the anti-colorectal cancer effect. Our data supported the anticancer potential of GNA as a component of therapeutic strategies for colorectal cancer.

## Data Availability Statement

The original contributions presented in the study are included in the article/Supplementary Material, further inquiries can be directed to the corresponding authors.

## Ethics Statement

The animal study was reviewed and approved by the Animal Experiment Centre of Shanghai University of Traditional Chinese Medicine.

## Author Contributions

CL, JX, and CG performed all the experiments. CL, JX, CG, XC, and CQ analyzed the data. CL wrote the manuscript. XZ, PZ, and YY read and approved the final version of the manuscript. All authors contributed to the article and approved the submitted version.

## Conflict of Interest

The authors declare that the research was conducted in the absence of any commercial or financial relationships that could be construed as a potential conflict of interest.

## Publisher’s Note

All claims expressed in this article are solely those of the authors and do not necessarily represent those of their affiliated organizations, or those of the publisher, the editors and the reviewers. Any product that may be evaluated in this article, or claim that may be made by its manufacturer, is not guaranteed or endorsed by the publisher.
