# Supplementary material for: Gambogenic Acid Induces Endoplasmic Reticulum Stress in Colorectal Cancer via the Aurora A Pathway
Source: Front Cell Dev Biol. 2021 Oct 6;9:736350. doi: 10.3389/fcell.2021.736350 (PMC8526855; doi:10.3389/fcell.2021.736350)
Supplement: Supplementary file 1 [file Data_Sheet_1.docx]

**Supplementary methods**

**Intracellular calcium measurement**

HCT116 cells were incubated in Fura 2-AM (5 μm) containing Ca^2+^ for 30 min in the dark. Working solution without Fura 2-AM was used to wash the cells, which were then treated with GNA at 37 ℃ for 50 s, and thapsigargin was added. Fluorescence was measured by an F4500 fluorescence spectrophotometer (Hitachi, Tokyo, Japan).

**SiRNA transfection**

Bip siRNA (5’-GAGGCUUAUUUGGGAAAGATT-3’, 5’-UCUUUCCCAAAUAAGCCUCTT-3’) and scramble control siRNA (5’-UUCUCCGAACGUGUCACGUdTdT-3’, 5’-ACGUGACACGUUCGGAGAAdTdT-3’) were purchased from GenePharma (Shanghai, China). Lipofectamine 2000 was used for cell transfection following the manufacturer’s instructions. **Cellular thermal shift assay**

HCT116 cells were treated with GNA (10 μM) for 1 h, added same volume of DMSO to another culture dish as the negative control. Cells suspended in cold PBS contained protease inhibitor. Distribute cells into ten different 0.2 ml tubes with 100 μl of cell suspension. Tubes were individually heated for 3 min at different temperatures ranging from 40 ℃ to 58 ℃, removed and incubated at room temperature for 3 min. After 3 min incubation, immediately snap-freeze the cells in liquid nitrogen. The cells were lysed by two freezed-traw cycles used liquid nitrogen, centrifuged at 20000×g for 20 min at 4 ℃. All samples were analyzed by western blotting.

**Supplementary Fig. 1GNA induced apoptosis in HCT116 cells.** Cells were treated with GNA for

24 h, and the ultrastructure of cells was observed under TEM after staining with uranyl acetate and

lead citrate (scale bar=2 μm). The data are presented for at least three independent experiments.

**Supplementary Fig. 2 GNA did not change intracellular calcium levels.** After loading with Fura

2-AM for 30 min, Ca^2+^ mobilization was analysed using a fluorescence spectrophotometer. GNA

was added 50 s after the monitoring began, and then thapsigargin was added 50 s for monitoring.

The data are presented for at least three independent experiments.

**Supplementary Fig. 3 GNA activated ER stress-associated apoptosis independent of PERK.** HCT116 cells treated with GNA (1 μM) for 24 h after preconditioning with 4-PBA (2 mM) and GSK2606414 (10 μM). Cell viability was assessed using an MTT assay. MTT solution was added to the well and incubated for 4 h at 37 °C. The precipitate was dissolved in DMSO, and the absorbance was measured at 570 nm.

**Supplementary Fig. 4 GNA activated ER stress-associated apoptosis independent of Bip** Bip siRNA was transfected into cells, (A) the expression of Bip was analysed by western blotting. (B, C) HCT116 cells treated with GNA (1 μM) after Bip knockdown for 24 h were fixed in 70% ethanol at 4 ℃, and flow cytometry was performed to examine the cell cycle after PI staining. The data are presented for at least three independent experiments.

**Supplementary Fig. 5 GNA interacts with Aurora A *in vitro*.** HCT116 cells treated with GNA (10 μM) for 1 h, cellular thermal shift assay was performed using HCT116 cells. The stabilization effect of GNA on Aurora A at different temperatures was determined by western blotting.

**Supplementary Fig. 6 The antitumour effect of GNA in vivo.** A colitis-associated cancer (CAC)

model was established. (A) Mice were administered AOM intraperitoneally and 2% DSS for 5 days,

followed by 16 days of regular water. (B) Changes in colon length after mice were sacrificed
