# Supplementary figures and images for: Gambogenic Acid Induces Endoplasmic Reticulum Stress in Colorectal Cancer via the Aurora A Pathway
Source: Front Cell Dev Biol. 2021 Oct 6;9:736350. doi: 10.3389/fcell.2021.736350 (PMC8526855; doi:10.3389/fcell.2021.736350)

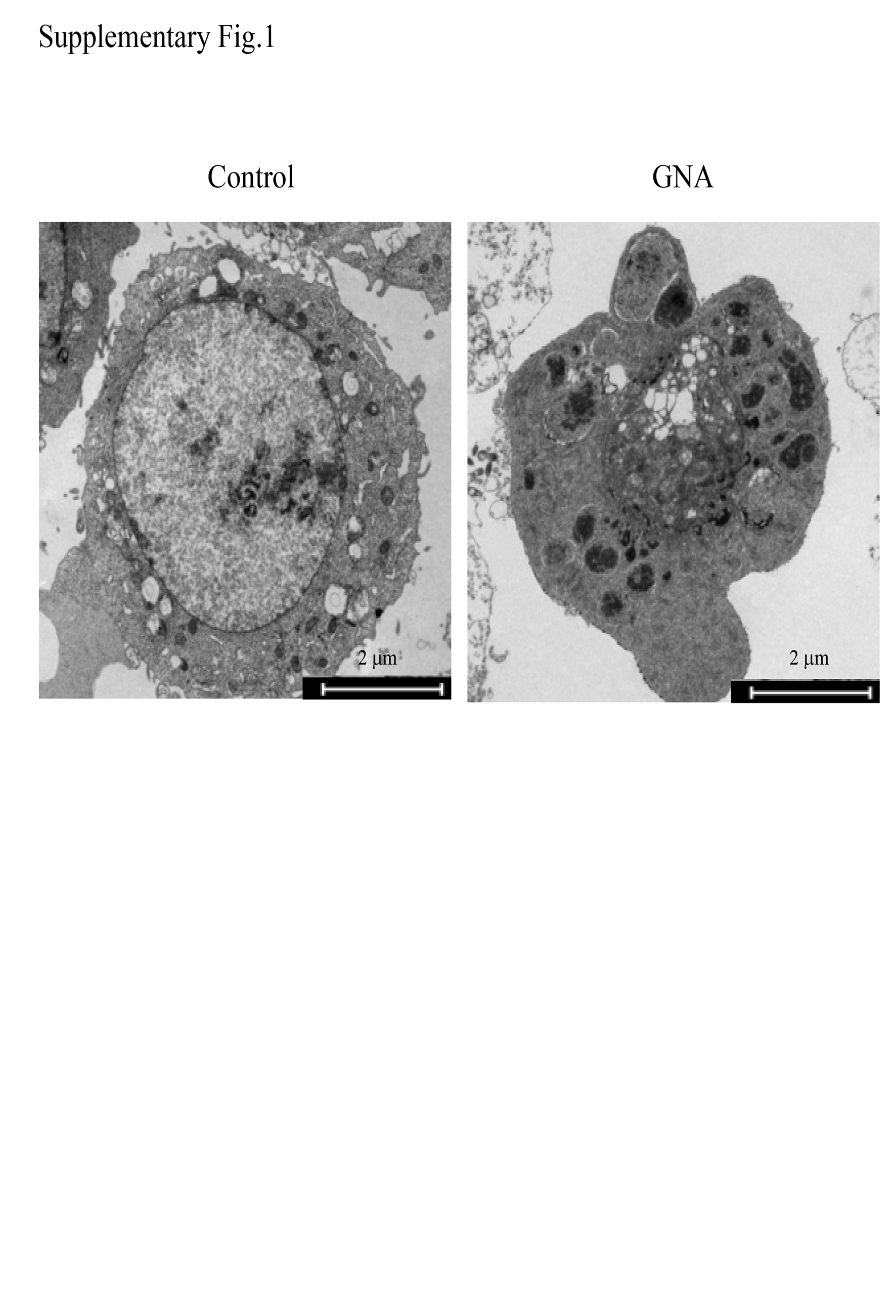

Supplement: Supplementary file 2 [file Image_1.TIF]

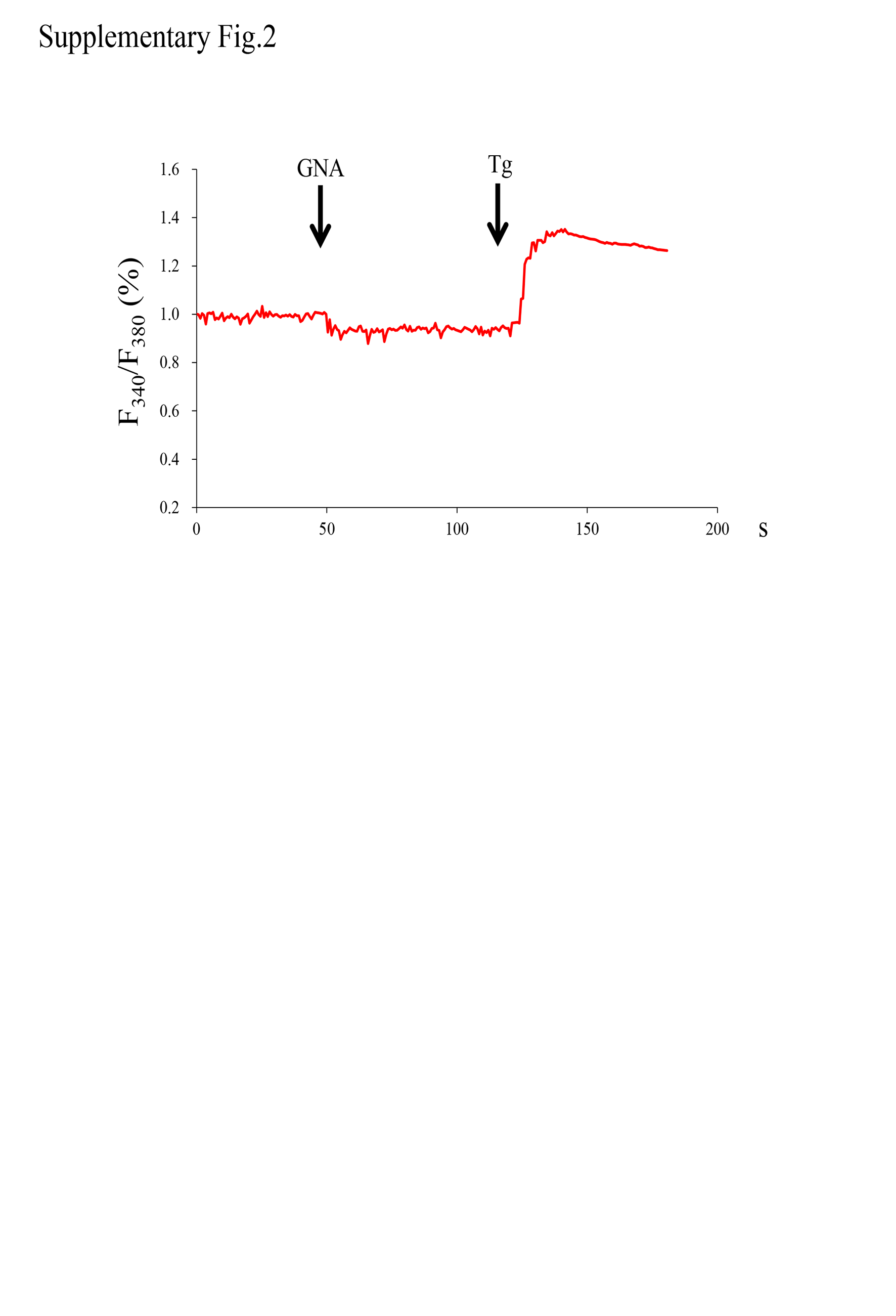

Supplement: Supplementary file 3 [file Image_2.TIF]

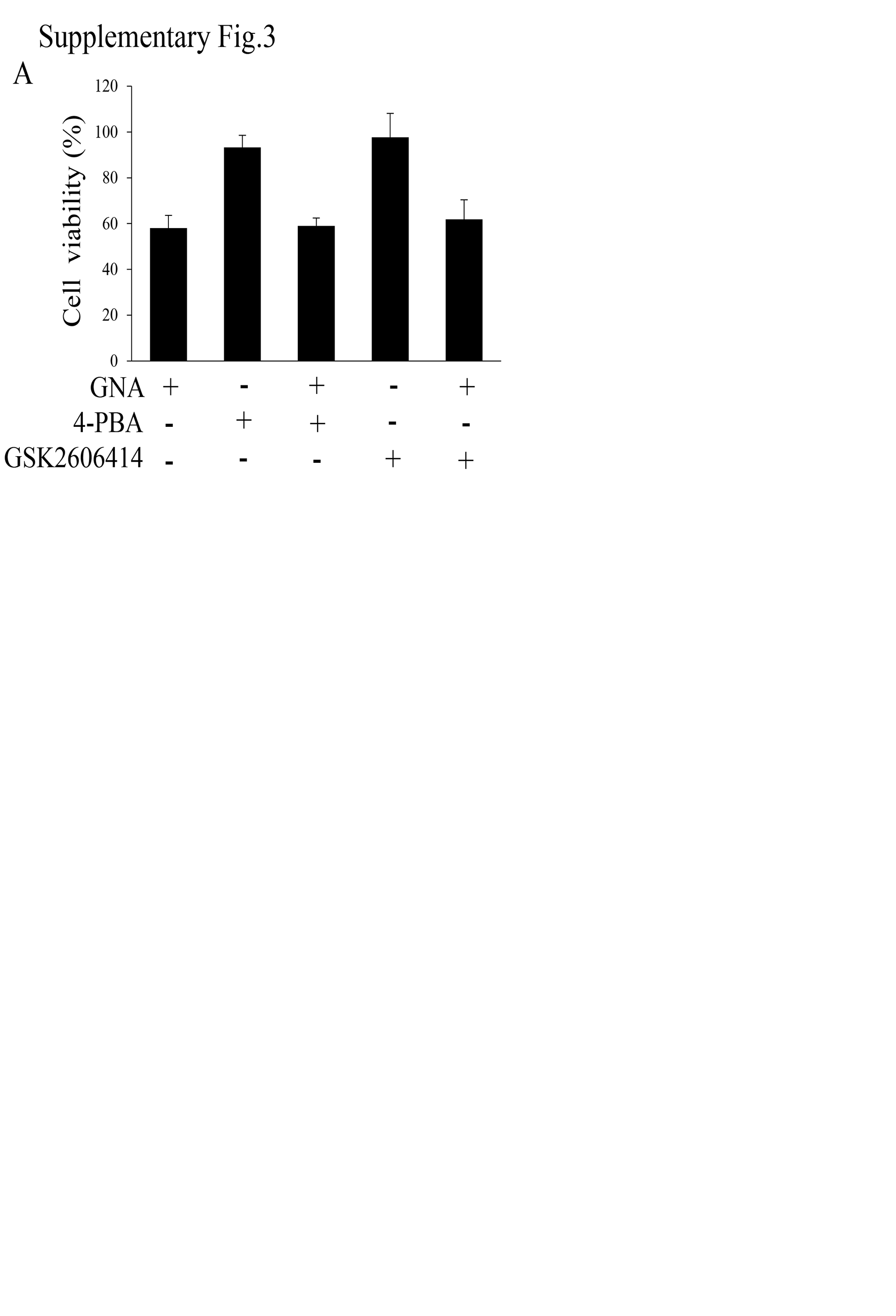

Supplement: Supplementary file 4 [file Image_3.TIF]

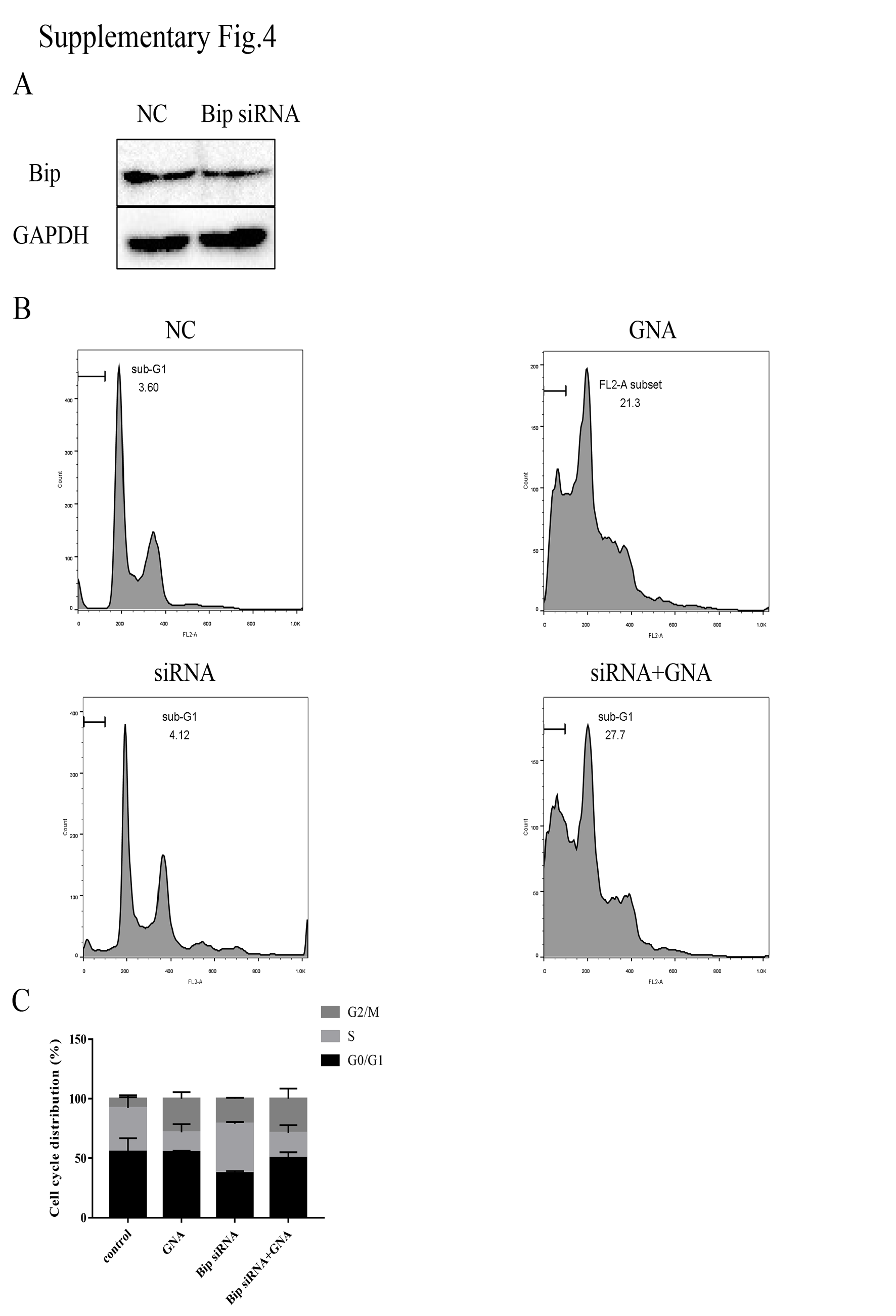

Supplement: Supplementary file 5 [file Image_4.TIF]

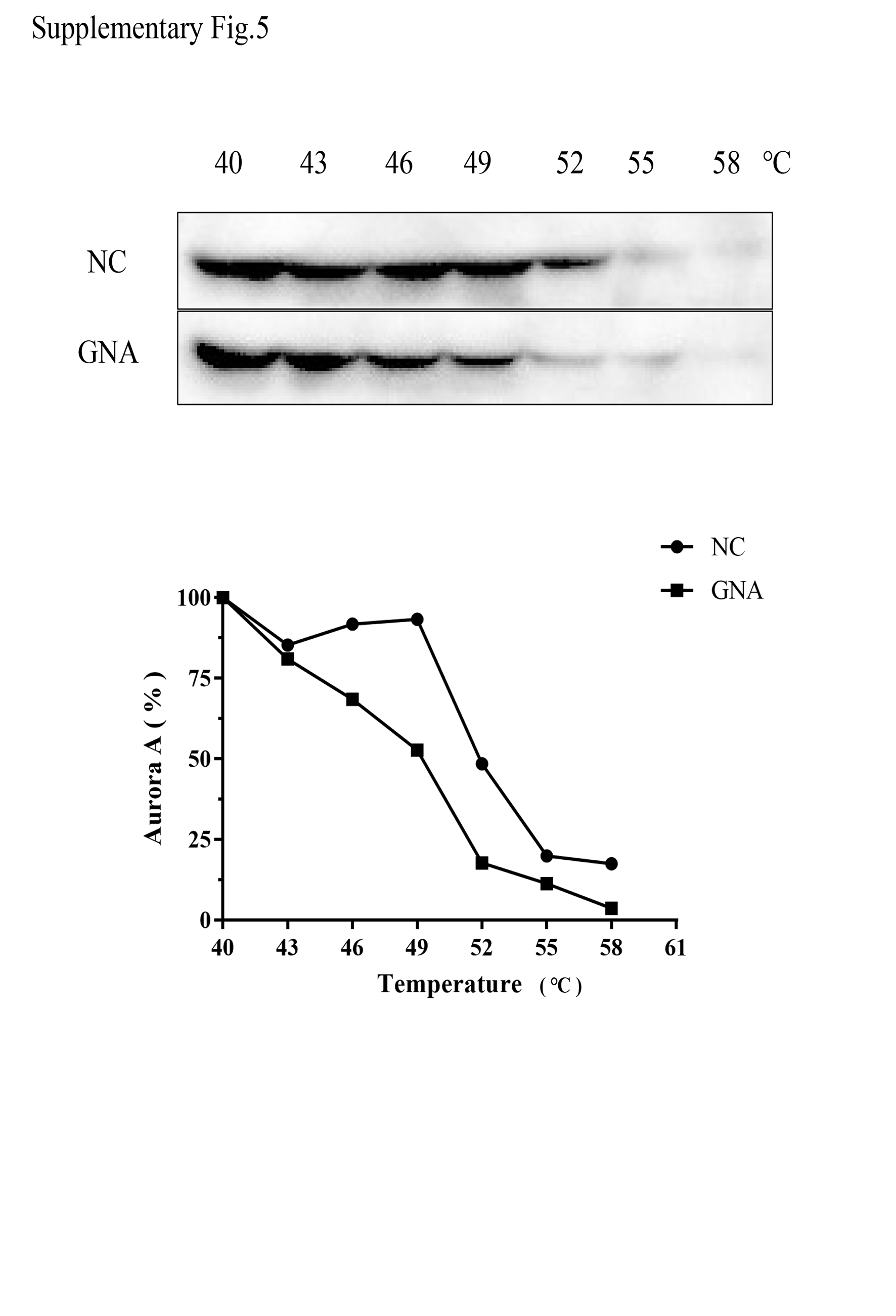

Supplement: Supplementary file 6 [file Image_5.tif]

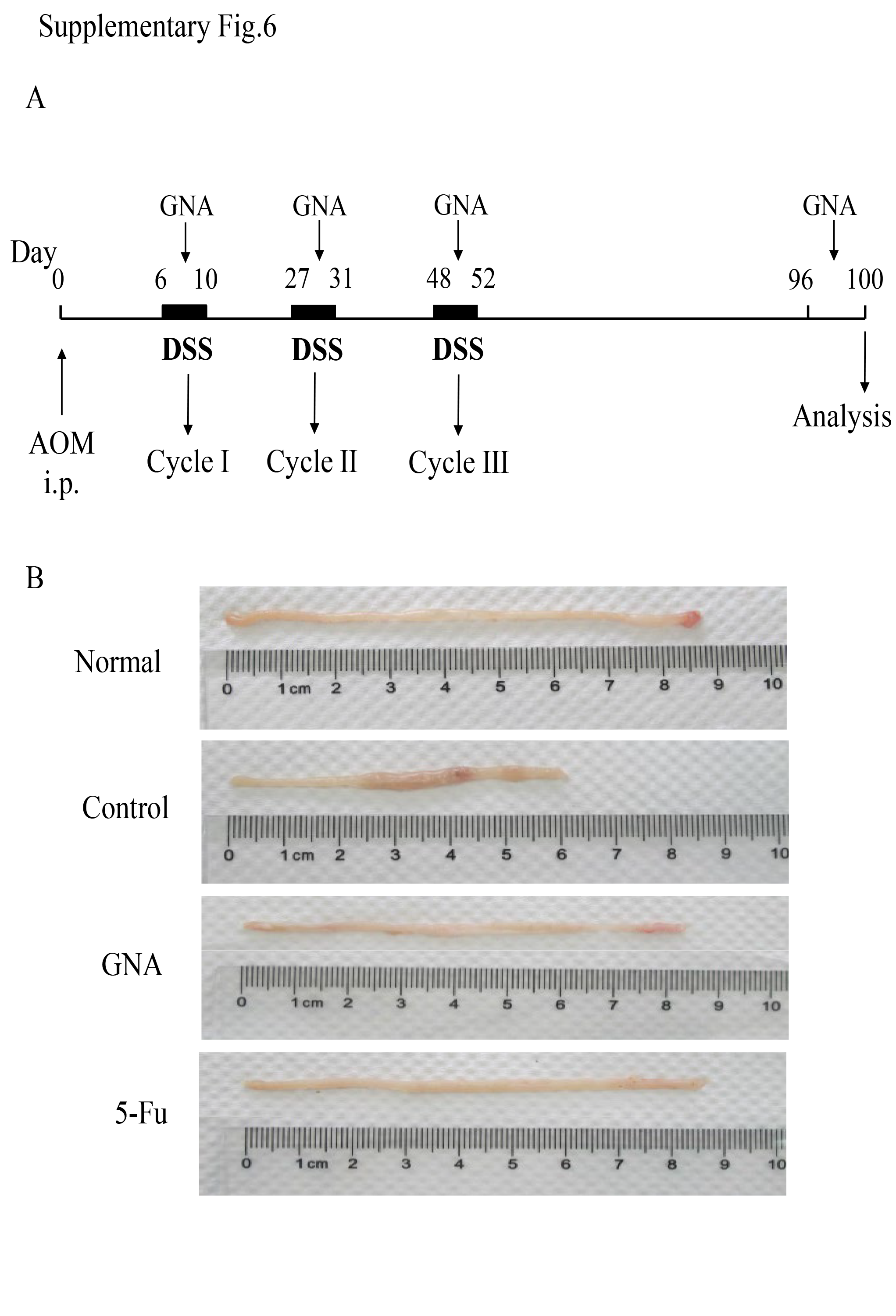

Supplement: Supplementary file 7 [file Image_6.TIF]
