# Supplementary material for: Gambogenic Acid Induces Endoplasmic Reticulum Stress in Colorectal Cancer via the Aurora A Pathway
Source: Front Cell Dev Biol. 2021 Oct 6;9:736350. doi: 10.3389/fcell.2021.736350 (PMC8526855; doi:10.3389/fcell.2021.736350)
Supplement: Supplementary file 8 [file Table_1.DOCX]

**Supplementary table 1 Primer sequences for real-time PCR**

| Gene | Primer (5′–3′) |
| --- | --- |
| Human-Bip-F | TGCTTGATGTATGTCCCCTTA |
| Human-Bip-R | CCTTGTCTTCAGCTGTCACT |
| Human-IREα-F | GCAGCAGACTTTGTCATCGG |
| Human-IREα-R | GTGATCACACACTCCCCCTTGT |
| Human-PERK-F | TGTCGCCAATGGGATAGTGACGAA |
| Human-PERK-R | AATCCGGCTCTCGTTTCCATGTCT |
| Human-eIF2-F | GAAGAGTGTGTTGGGCAGGT |
| Human-eIF2-R | TGGCTAGCAATCATGGCACT |
| Human-ATF4-F | TGGCCAAGCACTTCAAACCT |
| Human-ATF4-R | GTTGTTGGAGGGACTGACCAA |
| Human-ATF6-F | AGACTGAAGAGCAGGTGAGCAAA |
| Human-ATF6-R | GATGATGAAAAATGGAGCAGCTT |
| Human-CHOP-F | CAGAGCTGGAACCTGAGGAG |
| Human-CHOP-R | TGGATCAGTCTGGAAAAGCA |
| Human-GAPDH-F | CATCACCATCTTCCAGGAGCG |
| Human-GAPDH-R | TGACCTTGCCCACAGCCTTG |
